# Supplementary material for: Five Years Outcomes and Predictors of Events in a Single-Center Cohort of Patients Treated with Bioresorbable Coronary Vascular Scaffolds
Source: J Clin Med. 2020 Mar 20;9(3):847. doi: 10.3390/jcm9030847 (PMC7141535; doi:10.3390/jcm9030847)
Supplement: Supplementary file 1 [file jcm-09-00847-s001.pdf]

FIVE YEARS OUTCOMES AND PREDICTORS OF EVENTS IN A SINGLE-CENTER COHORT OF PATIENTS TREATED WITH BIORESORBABLE VASCULAR SCAFFOLDS

Remzi Anadol[1], Annika Mühlenhaus[1], Ann-Kristin Trieb[1], Alberto Polimeni[1,2], Thomas Münzel[1], Tommaso Gori[1]

[1]Kardiologie I, Zentrum für Kardiologie, University Medical Center Mainz and Deutsches Zentrum für Herz und Kreislauf Forschung, Standort Rhein-Main, Germany; [2] Division of Cardiology, Department of Medical and Surgical Sciences, "Magna Graecia" University, Catanzaro, Italy.

Supplemental material

	All patients (n=512)
Age (years) (Median, IQR)	62 (54-73)
Male	403 (78.7%)
Hypertension	362 (70.7%)
Diabetes	102 (19.9%)
Smoking	218 (42.6%)
Family History	100 (19.5%)
Dyslipidemia	190 (37.1%)
Prior CABG	11 (2.1%)
Prior PCI	135 (26.4%)
Prior stroke/TIA	17 (3.3%)
eGFR, (ml/min)	83 (69-99.5)
LVEF (%)	55 (50-55)
Silent/Stable angina	166 (32.4%)
Unstable angina	62 (12.1%)
NSTEMI	151 (29.5%)
STEMI	130 (25.4%)
ACS	346 (67.6%)
Clopidogrel	159 (31.1%)
Prasugrel	246 (48.0%)
Ticagrelor	107 (20.9%)
Follow up days<br>Median (IQR)	1868<br>(1640,5-2024)
Completed Follow up time	391 (76.4%)
Lesion characteristics (n=598 lesions)	
LAD treated with BRS	268 (44.8%)
LCX treated with BRS	156 (26.1%)
RCA treated with BRS	173 (28.9%)
Graft treated with BRS	1 (0.2%)
Ostial lesion	52 (8.7%)

**CTO**	17 (2.8%)
**Bifurcation**	79 (13.2%)
**B2 or C type lesion**	247 (41.3%)

Supplemental Table 1. Patient and lesion characteristics

Procedural characteristics	
Number of vessels treated with BVS per patient	1.2±0.5
BVS per patient	1.4±0.9
Hybrid BVS+DES (per patient)	305 (51.0%)
Predilatation	588 (98.7%)
Minimum stent diameter per patient (mm)	3.0 (2.5-3.0)
Total implanted length per patient (mm)	18 (18-30)
Postdilatation	210 (35.1%)
Sizing	1.0 (0.9-1.1)
Angiographic outcome	
% residual stenosis per lesion (%)	78 (67.5-100)
Reference Vessel diameter (RVD) pre implantation (mm)	2.905 (2.5-3.3)
RVD post (mm)	2.9 (2.6-3.3)
Scaled stenosis (%)	15.4 (6.7-24)
MLD pre	0.6 (0-0.9)
MLD post	2.52 (0.49)
MLD post/nominal BRS diameter per lesion	0.8 (0.7-0.9)
Footprint	37 (34-43)
"Optimal implantation" in lesion	214 (35.8%)
"Optimal implantation" per patient	205 (40.0%)
Overlap	69 (11.5%)

Supplemental Table 2. Procedural and post-procedural angiographic data

Covariate	HR	95% CI of HR	P
**Male**	356016.1937	7.31E-161 to 1.73E+171	0.9479
**Age**	0.9614	0.9204 to 1.0042	0.0783
**Hyperlipidemia**	0.7794	0.2415 to 2.5153	0.6783
**Hypertension**	2.2034	0.4923 to 9.8626	0.3040
**Diabetes**	**7.5041**	**2.4679 to 22.8174**	**0.0004**
**Smoking**	1.6798	0.5677 to 4.9709	0.3512
**Positive Family History**	1.1501	0.3185 to 4.1526	0.8318
**Prior CABG**	3.4311	0.4500 to 26.1635	0.2366
**Prior PCI**	1.7961	0.5908 to 5.4600	0.3044
**Prior stroke/TIA**	0.0000	4.29E-171 to 165E+159	0.9571
**eGFR**	**0.9702**	**0.9468 to 0.9941**	**0.0155**
**LVEF**	0.9812	0.9253 to 1.0404	0.5274
**Silent or stable angina**	0.8828	0.2735 to 2.8494	0.8357
**Unstabe angina**	0.6185	0.0813 to 4.7067	0.6443
**NSTEMI**	1.1268	0.3490 to 3.6375	0.8426
**STEMI**	1.2805	0.3966 to 4.1343	0.6808
**ACS**	1.1327	0.3510 to 3.6559	0.8357
**LAD treated**	0.9539	0.3223 to 2.8227	0.9323
**LCX treated**	1.2591	0.3900 to 4.0649	0.7015
**RCA treated**	0.8288	0.2296 to 2.9912	0.7754
**Ostial lesion**	0.0000	7.50E-260 to 149E+246	0.9664
**bifurcation**	0.4617	0.0607 to 3.5130	0.4577
**CTO**	0.0000	1.66E-221 to 43.93E+210	0.9671
**Complex lesion**	2.4970	0.8214 to 7.5901	0.1085
**Number of vessels treated**	1.0425	0.2995 to 3.6293	0.9481
**Number of BVS**	1.1574	0.6264 to 2.1385	0.6425
**Hybrid stenting**	0.6399	0.2162 to 1.8935	0.4223
**Predilatation**	13456	4.27E-211 to 423.51E+216	0.9701
**Minimum stent diameter per patient**	1.3206	0.3121 to 5.5871	0.7070
**Total length of scaffold**	1.0038	0.9755 to 1.0329	0.7970
**Post dilatation**	0.1645	0.0216 to 1.2519	0.0829
**sizing**	10.6552	0.2045 to 555.2400	0.2432
**Residual % stenosis**	1.0184	0.9640 to 1.0758	0.5171
**RVD**	0.9479	0.3220 to 2.7909	0.9231
**RVD<2.5**	1.0256	0.2286 to 4.6020	0.9738
**RVD>3.5**	0.6376	0.0832 to 4.8866	0.6665
**RVD post implantation**	0.7159	0.2244 to 2.2838	0.5743
**Scaled stenosis**	1.0327	0.9872 to 1.0803	0.1637
**MLD**	3.2057	0.9610 to 10.6939	0.0594
**MLD post implantation**	0.6310	0.1926 to 2.0678	0.4494
**MLD/nominal**	0.2382	0.0135 to 4.1910	0.3293
**Maximum footprint**	1.0314	0.9595 to 1.1086	0.4041
**Overlap**	0.7876	0.1036 to 5.9893	0.8185
**clopidogrel**	0.6936	0.1921 to 2.5036	0.5783
**prasugrel**	1.7280	0.5685 to 5.2522	0.3373
**ticagrelor**	0.6549	0.1463 to 2.9320	0.5819
**Optimal Implantation**	0.9070	0.2984 to 2.7569	0.8641

Supplemental Table 3. Predictors of TLF in univariate cox regression analysis 4-5 years after implantation.

Multivariate Cox regression model

	HR	p
**Diabetes**	**6.21(1.99-19.40)**	**0.002**
eGFR	0.98(0.95-1.00)	0.08

Supplemental Table 4. Predictors of TLF in univariate and multivariate cox regression analysis 4-5 years after implantation
